# SynergyFinder 3.0: an interactive analysis and consensus interpretation of multi-drug synergies across multiple samples

**DOI:** 10.1093/nar/gkac382

**Published:** 2022-05-17

**Authors:** Aleksandr Ianevski, Anil K Giri, Tero Aittokallio

**Affiliations:** Institute for Molecular Medicine Finland (FIMM), HiLIFE, University of Helsinki, Finland; Helsinki Institute for Information Technology (HIIT), Aalto University, Finland; Institute for Molecular Medicine Finland (FIMM), HiLIFE, University of Helsinki, Finland; Foundation for the Finnish Cancer Institute (FCI), University of Helsinki, Finland; Institute for Molecular Medicine Finland (FIMM), HiLIFE, University of Helsinki, Finland; Helsinki Institute for Information Technology (HIIT), Aalto University, Finland; Institute for Cancer Research, Department of Cancer Genetics, Oslo University Hospital, Norway; Centre for Biostatistics and Epidemiology (OCBE), Faculty of Medicine, University of Oslo, Norway

## Abstract

SynergyFinder (https://synergyfinder.fimm.fi) is a free web-application for interactive analysis and visualization of multi-drug combination response data. Since its first release in 2017, SynergyFinder has become a popular tool for multi-dose combination data analytics, partly because the development of its functionality and graphical interface has been driven by a diverse user community, including both chemical biologists and computational scientists. Here, we describe the latest upgrade of this community-effort, SynergyFinder release 3.0, introducing a number of novel features that support interactive multi-sample analysis of combination synergy, a novel consensus synergy score that combines multiple synergy scoring models, and an improved outlier detection functionality that eliminates false positive results, along with many other post-analysis options such as weighting of synergy by drug concentrations and distinguishing between different modes of synergy (potency and efficacy). Based on user requests, several additional improvements were also implemented, including new data visualizations and export options for multi-drug combinations. With these improvements, SynergyFinder 3.0 supports robust identification of consistent combinatorial synergies for multi-drug combinatorial discovery and clinical translation.

## Graphical Abstract

**Graphical Abstract:**
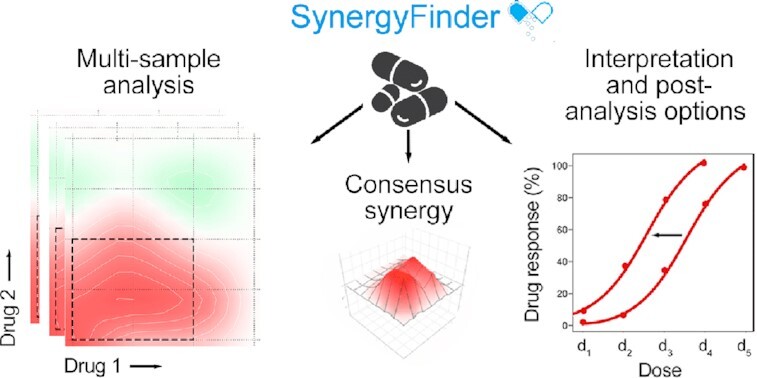
SynergyFinder 3.0: an interactive analysis and consensus interpretation of multi-drug synergies across multiple samples.

## INTRODUCTION

Combination therapies are used to treat patients with hypertension, HIV, tuberculosis, COVID-19 and many drug-resistant cancers (1–6). Multi-drug treatments can result in therapeutic benefits both by enhancing the treatment efficacy and by avoiding the acquisition of monotherapy resistance (7–10). Historically, drug combinations have been identified using a trial-and-error method that requires considerable time and may lead to sub-optimal results (7,11,12). High-throughput screening (HTS) technologies have enabled a more systematic and accelerated discovery of new drug combination candidates (4,9,13–15). With HTS, thousands of drugs combinations can be tested in multiple doses in preclinical model systems to identify synergistic drug combinations, i.e. combinations that result in a higher-than-expected effect. The expected effect of drug combinations can be estimated mathematically, using a reference or null model, which quantifies the expected combination effect under the null hypothesis of no interaction between the single-agents (10).

To facilitate the discovery of synergistic combinations, several freely available software tools have emerged for the analysis of high-throughput combinatorial screening data (15–21). Most of the tools implement multiple reference models, including Bliss excess (22), Loewe additivity (23), highest single-agent (HSA) (24) and zero interaction potency (ZIP) (25) for synergy scoring. However, since these models are formulated under rather different assumptions of single-drug behaviour (26), a careful interpretation of the identified synergy and antagonism patterns is essential for avoiding false positive and negative findings. For instance, one may end up in different interpretations of synergy when using different synergy scoring models, and the users may therefore easily become biased towards selection of a reference model that best supports their hypotheses. Such ‘multiple testing bias’ may hamper consistency between synergy studies, lead to delays in the discovery of true synergistic drug combinations, and negatively impact the translatability of combination discovery efforts (27).

Moreover, measurement errors in single-drug dose-response data may also lead to biased synergy estimation and false interpretation of the drug testing results, under different synergy models, unless suitable analytic options are provided for the users (26,28,29). For instance, reporting of low-confidence synergy that originate from outliers in single-agent responses may lead to inconsistent or incorrect combination discoveries (28). Together with the fact that a synergy between drugs is very much dependent on the cell-context (e.g. cell line or patient sample) and drug-class (e.g. targeted versus non-targeted therapy), these issues are contributing to the reproducibility crisis in the field of pre-clinical cancer drug development, leading to alarming reports that the primary findings are hard to replicate in *in vivo* pre-clinical studies and in the clinical setting (30). Therefore, easy-to-use software solutions that allow for an unbiased and robust *in silico* prioritization of synergistic combinations bases on *in vitro* or *ex vivo* drug combination testing are indispensable for successful therapeutic development.

To match these needs, we have implemented SynergyFinder web-application version 3.0, which enables simultaneous analysis and interactive visualization of drug combinations assessed with multiple synergy reference models in multiple doses and samples. Such multi-sample and multi-model analysis considers the context-dependency of synergies, reduces the risk of reporting false synergies due to experimental errors, and enables the users to better interpret the drug combination results. In particular, an interactive multi-sample analysis provides the users with an improved means to perform a comparative analysis of combination synergy profiles across multiple samples and patient groups for more robust statistical conclusions. Finally, novel post-analysis options improve the interpretation of the results by allowing one to highlight combinations that show synergy at lower dose windows, which are less likely to result in toxic responses, as well as to distinguish between different modes of synergy (potency and efficacy, 31) to better rationalize the drug combination development.

## RESULTS

### An overview of the extended functionality of SynergyFinder 3.0

SynergyFinder provides a complete pipeline for drug combination synergy assessment based on multi-dose experimental assays (18,20). Designed to be accessible by researchers with little or no programming skills, SynergyFinder web-application requires only the experimental testing data as an input (e.g. percentage inhibition compared to control), and it enables several options for pre-processing and automated synergy analyses, thereby significantly reducing the time required for manual analysis of large-scale combinatorial screening experiments. In addition to supporting HTS efforts, the web-app is also applicable to analysing data from more targeted combination testing, even from individual combinations. The web-tool provides various interactive plots and summary statistics, and it allows for exporting publication-quality figures and reports of the combination data and synergy results.

The new SynergyFinder version 3.0 implements: (i) a multi-sample synergy analysis and interactive visualizations that enable simultaneous analysis of drug combinations tested in various samples and doses, (ii) a novel summary metric that unifies multiple synergy reference models and an automated outlier replacement in single-drug response measurements to eliminate false-positive results, (iii) a post-analysis option that enables weighting of synergy by concentrations to highlight combinations that show synergy at lower dose windows and are less likely to lead to toxic responses in clinical application, (iv) another post-analysis option that allows for distinguishing between different synergy types (e.g. potency and efficacy), using a recently-developed parametric synergy model, MuSyC (31,32) and (v) new interactive visualization options that enhance the interpretation of combination synergies.Additional improvements requested by the users include the possibility to use as input also count data (e.g. the number of cancer cells killed in treatment), as an alternative to % inhibition, as well as enhanced interactive options, e.g. custom selection of most synergistic area, adjustable colour bars, and many more.

### Multi-sample synergy analysis and interactive heatmap visualizations

**Figure 1.**
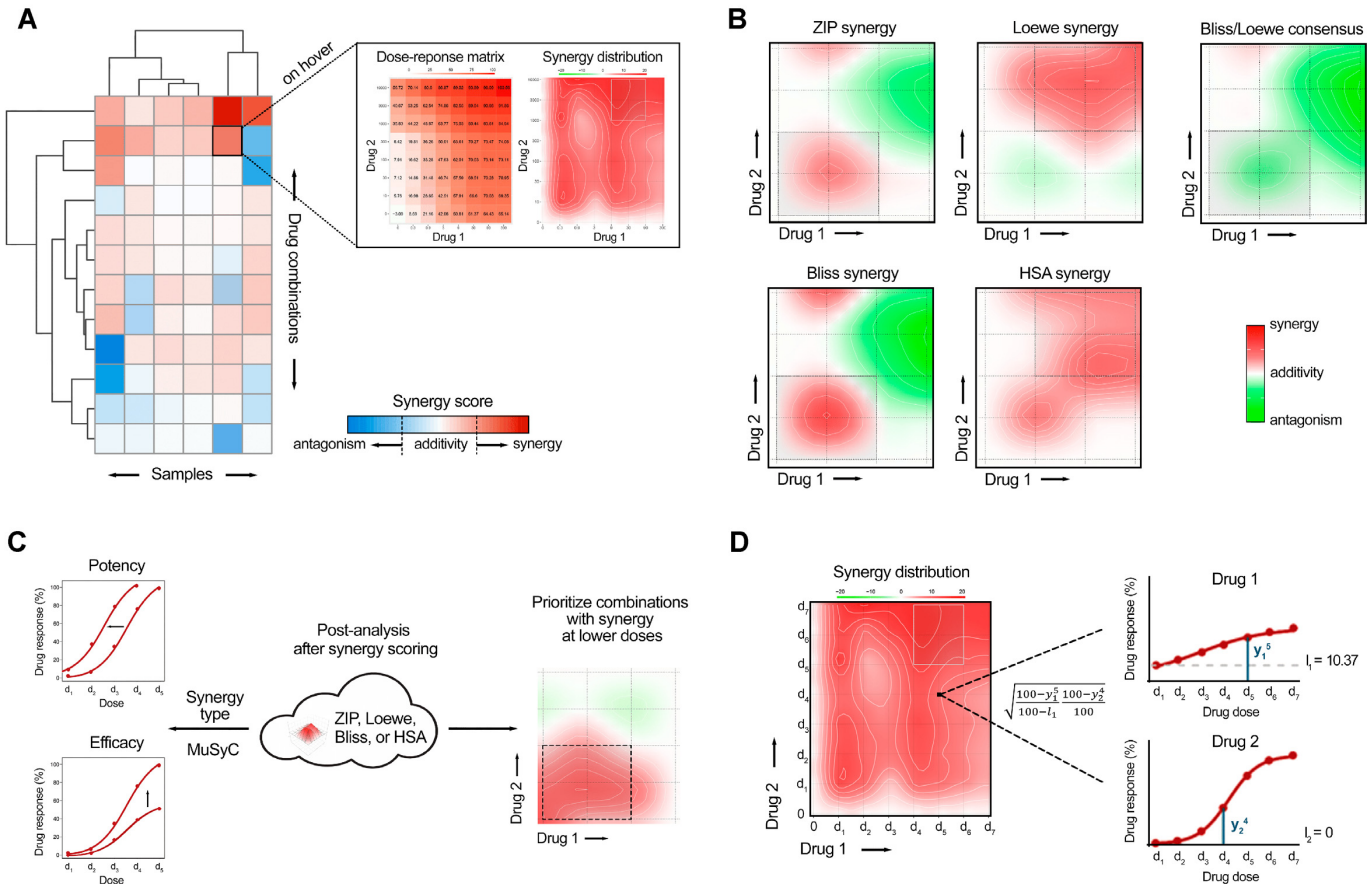
Novel features implemented in the SynergyFinder version 3.0 include (**A**) multi-sample analysis with interactive visualization of combination effects across the samples, (**B**) Bliss/Loewe consensus scoring to eliminate false positive synergy results (in the example case, user-selected HSA model identifies moderate-to-strong synergy, whereas Bliss/Loewe consensus suggests a strong antagonism, indicating low-confidence HSA synergy), (**C**) multiple post-analysis options, such as distinguishing between different modes of synergy (potency and efficacy), and prioritizing of combinations that show maximal synergy at lower doses (the dotted box). (**D**) An example of how SynergyFinder highlights combinations that show synergy at lower dose windows by weighting synergy at each dose level according to the proportion of response values of single-agents at these doses (see text for details).

Currently, there are no software tools available that would enable systematic analysis and visualization of drug combination synergy across multiple samples in medium- or high- throughput combinatorial experiments. SynergyFinder 3.0 enables such visualizations using an interactive heatmap that allows for a multi-sample analysis and supports the identification of both common and context-specific synergies that occur across multiple samples (Figure 1A); for instance, shared dependencies on sample-specific molecular features, such as mutations, or unique sample-specific synergies that are often observed in cancer patients (5). While the SynergyFinder 2.0 focused more on statistical treatment of experimental replicates (20), the newest version 3.0 makes it possible to leverage information from multiple independent samples to consider biological variability in combination synergy.

An example of a multi-sample interactive heatmap visualization can be found at https://synergyfinder.fimm.fi/synergy/heatmap.html, which shows the multi-sample analysis results of the DECREASE anticancer combination dataset (15). SynergyFinder also implements an interactive waterfall plot that shows synergy results for individual samples (both dose-response matrix and synergy distribution), and which can be generated even for single-sample input data (see example https://synergyfinder.fimm.fi/synergy/waterfall.html). Integrating the sample-specific synergy scores with multi-omics information available from the samples is expected to help statistical downstream analyses, e.g. to explain the observed variability in synergy patterns across either independent patient samples or cell lines, which can lead to novel genomics and molecular markers for combination effects.

### A novel consensus score and outlier detection for improved reproducibility

SynergyFinder 3.0 provides a novel synergy scoring method (called Bliss/Loewe consensus) that combines multiple synergy reference models (Bliss, Loewe, and HSA), both for pairwise and higher-order combination data. The ZIP synergy model was not included in the consensus scoring as it shares the same multiplicative survival principle as Bliss model, thereby potentially biasing the consensus synergy interpretation (see Supplementary Figure S1). More specifically, the web-app quantifies the expected combination effect based on all the three reference models, and then calculates a consensus distribution that is the maximum expected combination effect among the models at each drug combination dose pair (Figure 1B). Since the HSA synergy score always results in an expected effect equal or lower than that of Bliss and Loewe consensus, the combined score is called Bliss/Loewe consensus.

We note that even if some combinations are not consistently identified by both models as synergistic (using the Bliss/Loewe consensus score), they may still show true synergy that was not captured by these models. Therefore, the primary aim of such consensus scoring is to eliminate those false positive synergy cases, where a user has selected a potentially biased reference model that best supports the user's expectations. Supplementary Figure S2 shows an example of such potential false positive synergy detection with the HSA model. We recommend one to calculate consensus synergy score for all identified top-synergistic combinations, and then further investigate (and potentially de-prioritize) those that show Bliss/Loewe consensus synergy <–5, e.g. using the post-analysis options (see below).

In addition, researchers sometimes tend to report false synergies that are due to outliers in the combination measurements or single-agent dose-responses. In SynergyFinder 2.0 (20), we implemented an automated detection of outlier measurements using our machine learning model, built on the novel composite non-negative matrix factorization (cNMF) algorithm (15). The measured combination responses that deviate >20% inhibition from the cNMF predictions are marked as possible outlier measurements, and the users may replace them with the predicted values. In SynergyFinder 3.0, we have extended this functionality to automatically identify and replace outlier measurements also in single-drug dose-responses, with the same predefined cut-off difference of 20% inhibition, compared to control, thus minimizing the impact of single-agent outliers on the synergy calculations.

Such summary synergy scoring and outlier detection procedures are expected to improve the reproducibility, consistency and translatability of the combination discoveries.

### Post-analysis options for better interpretation of combination synergy results

In SynergyFinder 3.0, we have also implemented several post-analysis options that enable the users to explore and better interpret the identified synergistic combinations (Figure 1c). As the first post-analysis option, we incorporated into SynergyFinder 3.0 the recently-introduced parametric synergy scoring model, Multi-dimensional Synergy of Combinations (MuSyC), since it provides the users with the possibility to distinguish whether the identified synergy is due to enhanced potency and/or efficacy of the single agents; such post-scoring option should benefit both the anticancer and other disease applications (31). MuSyC model is also expected to provide a more consistent and unbiased interpretation of drug combinations, initially identified using the standard reference models, even though it generally assumes that the single-drugs in combinations have monotonic, sigmoidal dose-responses (32).

The second post-analysis option is to weight synergy by concentrations, hence highlighting combinations that show synergy at lower dose windows (Figure 1C, right), as those combinations are less likely to lead to toxic responses and are better tolerated by patients in the clinics. Since the tested drug concentration ranges are often heterogenous across the drugs in combination assays, SynergyFinder does not directly utilize drug doses, but rather the proportion of response values of single-agents at each dose. In this way, SynergyFinder implicitly favours synergy at lower concentrations without the need to utilize the absolute drug concentration levels. More specifically, the synergy distribution at each dose level is weighted by $\sqrt {\frac{{100 - y_1^i}}{{100 - {l_1}}}\frac{{100 - y_2^j}}{{100 - {l_2}}} \ldots \frac{{100 - y_n^m}}{{100 - {l_n}}}}$, where $y_n^m$ is the %inhibition response of drug *n* at dose *m*, and ${l_n}$ is the lower asymptote of the fitted dose-response curve for *n*th drug (Figure 1D).

## CONCLUSIONS

We have implemented SynergyFinder 3.0, a freely available web-application that enables the users to interactively process, assess, explore, visualize and report the most confident multi-drug synergies from multi-dose drug combination assays. By allowing the users to estimate the Bliss/Loewe consensus synergy, remove the low-confident synergy hits, and make a more detailed exploration of the mode of synergy and antagonism, SynergyFinder 3.0 provides a computational platform for fast, reliable and reproducible synergy scoring with interactive visualization options for multi-drug combinations, either from targeted, medium-throughput or high-throughput combination screens. The new features of the web-tool are expected to improve the consistency of drug combinations screens, accelerate the discovery of synergistic combinations, and enhance the translatability of discovered combinations into the clinic.

In particular, the novel multi-sample analysis option implemented in SynergyFinder 3.0 helps the users to evaluate both consistent and unique synergies for downstream biomarker identification, through integration of the synergy scores with other multi-omics data, thereby offering the possibility to further explore the determinants of synergy/antagonism and to improve the translatability of the finding from preclinical models (33). We recommend the use of multi-sample and multi-dose assays for more reliable synergy analyses in applications where access to excess sample material is available (e.g. patient cells). We further encourage users to continue leaving comments or suggestions for further improvements using the feedback form available on the website, as well as implement or request extended functionality through the GitHub repository, with the aim of making SynergyFinder even more interactive and user-friendly.

We believe that the upgraded SynergyFinder 3.0 web-platform will become even a more popular tool, enabling robust multi-drug and multi-sample synergy analyses with higher confidence, consistency, and interpretability, supporting many exciting applications of multi-drug combinatorial discovery and clinical translation.

## DATA AVAILABILITY

SynergyFinder is a freely available web-app hosted at https://synergyfinder.fimm.fi without any login requirements. The software comes with example drug combination data, video tutorial, and technical user guide and instructions available on the landing page. The source codes of the web-app are available at https://github.com/IanevskiAleksandr/SynergyFinder (under the BSD 3-clause license) to allow extension of the tool for further applications and integration with other software solutions.

## Supplementary Material

gkac382_Supplemental_File
