# Supplementary material for: SynergyFinder 3.0: an interactive analysis and consensus interpretation of multi-drug synergies across multiple samples
Source: Nucleic Acids Res. 2022 May 17;50(W1):W739–43. doi: 10.1093/nar/gkac382 (PMC9252834; doi:10.1093/nar/gkac382)
Supplement: gkac382_Supplemental_File [file gkac382_supplemental_file.pdf]

Supplementary data

SynergyFinder 3.0: an interactive analysis and consensus interpretation of multi-drug synergies across multiple samples

Aleksandr Ianevski[1,2], Anil K Giri[1,3*] and Tero Aittokallio[1,2,4,5*]

Further results on the Bliss/Loewe consensus scoring

To compare the Bliss/Loewe consensus score against the ZIP synergy score, we used the DECREASE combination dataset (http://decrease.fimm.fi/data_availability) to correlate the synergy scores calculated with ZIP and Bliss/Loewe consensus models (Fig. S1a), as well as with ZIP and Bliss models (Fig. S1b). This analysis demonstrates that the consensus model provides a non-orthogonal means to validate hits obtained using single scores, such as ZIP, while ZIP and Bliss scores are highly correlated (*R*=0.99, *p*<0.0001).

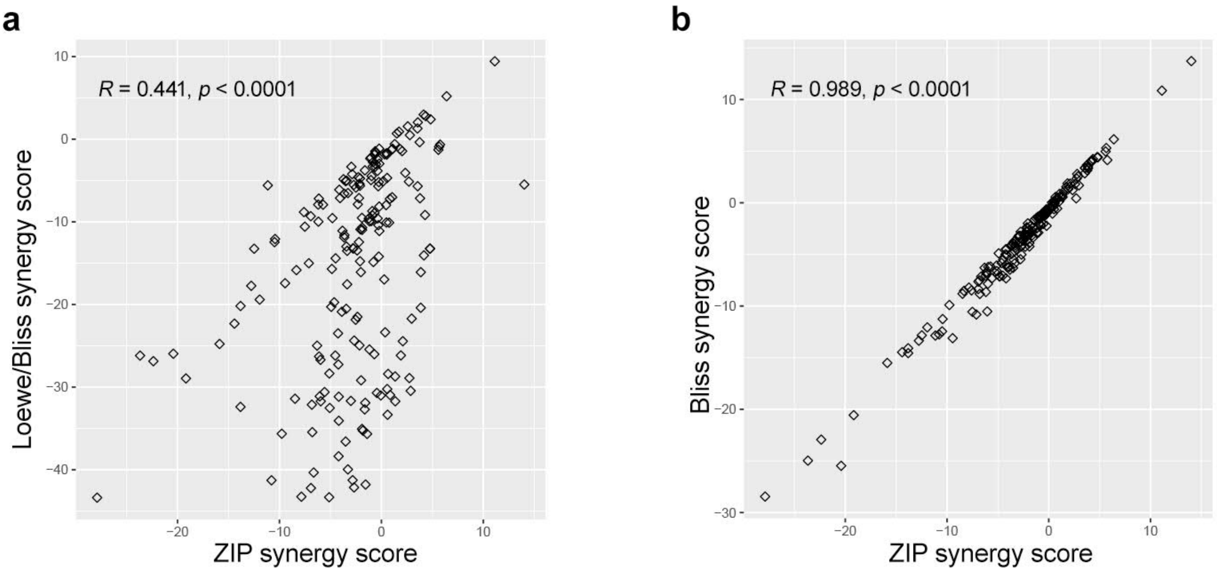

Figure S1. Relationship between the synergy scores calculated with ZIP and Bliss/Loewe consensus models **(a)**, as well as with ZIP and Bliss models **(b)** in the DECREASE combination dataset (http://decrease.fimm.fi/data_availability).

We further used the same DECREASE dataset to show an example, where the user may become biased towards the HSA synergy score and report a finding of a synergistic combination between Dactolisib-Everolimus (Fig. S2a), solely based on the HSA model (HSA synergy: 5.2, maximum synergistic area (MSA, dotted box): 13.4). However, the ZIP synergy score is -3.5, and the Bliss/Loewe consensus is -5.1, suggesting that the relatively strong synergy reported by HSA can be a false positive finding, and the combination should be investigated further (Fig S2b). Indeed, in the post-analysis investigation, no synergy for this combination was detected with 95% confidence interval by the MuSyC model.

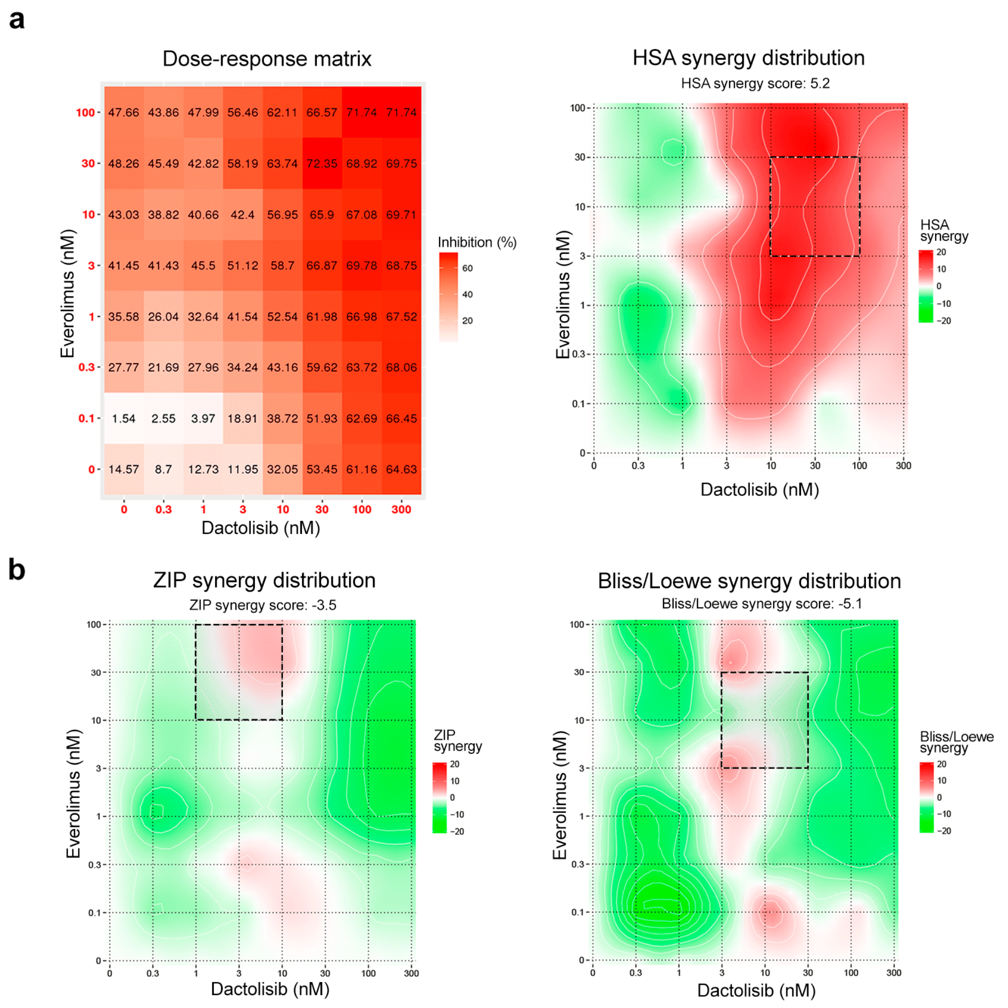

Figure S2. (a) An example of potential false positive synergistic combination identified by the the HSA model alone. **(b)** HSA, ZIP and Bliss/Loewe synergy scores for the combination shown in panel a. The dotted boxes correspond to the maximal synergistic area within each matrix.

We would like to note that the primary aim of such consensus scoring is to eliminate those false positive synergy cases, where a user has selected a potentially biased reference model that best supports the user's expectations. We recommend one to calculate consensus synergy score for all the identified top-synergistic combinations, and then further investigate (and potentially de-prioritize) those that show Bliss/Loewe consensus synergy < -5.
